# Supplementary material for: Spiny-Cheek Crayfish, Faxonius limosus (Rafinesque, 1817), as an Alternative Food Source
Source: Animals (Basel). 2020 Dec 30;11(1):59. doi: 10.3390/ani11010059 (PMC7823787; doi:10.3390/ani11010059)
Supplement: Supplementary file 1 [file animals-11-00059-s001.pdf]

Table S1. The accuracy of the analytical method verified against certified reference material: ERM-BB422 Fish muscle

Element	Certified value		Determined value (n = 3)		Recovery	RSD
	mg kg^{-1}	SD	mg kg^{-1}	SD	%	%
Ca	3420	*	3487	61	102	1.7
K	21400	*	21643	895	101	4.1
Mg	1370	*	1304	17	95	1.3
Na	2800	*	2937	91	105	3.1
Fe	9.4	1.4	9.0	0.3	96	3.3
Cu	1.67	0.16	1.69	0.09	101	5.3
Zn	16	1.1	16.8	0.8	105	4,8
Cd	0.0075	0.0018	0.0072	0.0008	96	11.1

* Information on SD for Ca, K, Mg and Na were not provided by the ERM provider. (SD – Standard Deviation, RSD – Relative Standard Deviation)
